# Photosynthetic Enhancement, Lifespan Extension, and Leaf Area Enlargement in Flag Leaves Increased the Yield of Transgenic Rice Plants Overproducing Rubisco Under Sufficient N Fertilization

**DOI:** 10.1186/s12284-022-00557-5

**Published:** 2022-02-09

**Authors:** Marin Tanaka, Mamoru Keira, Dong-Kyung Yoon, Tadahiko Mae, Hiroyuki Ishida, Amane Makino, Keiki Ishiyama

**Affiliations:** Graduate School of Agricultural Science, Tohoku University, 468-1 Aramaki-Aoba, Aoba-ku, Sendai, 980-8572 Japan

**Keywords:** Canopy architecture, Flag leaf, Grain yield, Lifespan, Light-reception, Nitrogen, Photosynthesis, *RBCS*-sense rice plants, Rubisco

## Abstract

**Background:**

Improvement in photosynthesis is one of the most promising approaches to increase grain yields. Transgenic rice plants overproducing Rubisco by 30% (*RBCS*-sense rice plants) showed up to 28% increase in grain yields under sufficient nitrogen (N) fertilization using an isolated experimental paddy field (Yoon et al. in Nat Food 1:134–139, 2020). The plant N contents above-ground sections and Rubisco contents of the flag leaves were higher in the *RBCS*-sense plants than in the wild-type rice plants during the ripening period, which may be reasons for the increased yields. However, some imprecise points were left in the previous research, such as contributions of photosynthesis of leaves below the flag leaves to the yield, and maintenance duration of high photosynthesis of *RBCS*-sense rice plants during ripening periods.

**Result:**

In this research, the photosynthetic capacity and canopy architecture were analyzed to explore factors for the increased yields of *RBCS*-sense rice plants. It was found that N had already been preferentially distributed into the flag leaves at the early ripening stage, contributing to maintaining higher Rubisco content levels in the enlarged flag leaves and extending the lifespan of the flag leaves of *RBCS*-sense rice plants throughout ripening periods under sufficient N fertilization. The higher amounts of Rubisco also improved the photosynthetic activity in the flag leaves throughout the ripening period. Although the enlarged flag leaves of the *RBCS*-sense rice plants occupied large spatial areas of the uppermost layer in the canopy, no significant prevention of light penetration to leaves below the flag leaves was observed. Additionally, since the CO_2_ assimilation rates of lower leaves between wild-type and *RBCS*-sense rice plants were the same at the early ripening stage, the lower leaves did not contribute to an increase in yields of the *RBCS*-sense rice plants.

**Conclusion:**

We concluded that improvements in the photosynthetic capacity by higher leaf N and Rubisco contents, enlarged leaf area and extended lifespan of flag leaves led to an increase in grain yields of *RBCS*-sense rice plants grown under sufficient N fertilization.

**Supplementary Information:**

The online version contains supplementary material available at 10.1186/s12284-022-00557-5.

## Background

By 2050, the world’s population is expected to reach 10 billion, and the United Nations Food and Agricultural Organization has predicted that the world population will need 70% more food (Long 2012). The rice yield increases after the “green revolution” depended mainly on the development of semidwarf cultivars with a greater harvest index and greatly increased N fertilizer application (Yoshida 1981; Evans 1998). This strategy is reaching its limits. Excess application of N fertilizer causes environmental pollution, including acid rain and the eutrophication of rivers and oceans (Canfield et al. 2010; Good and Beaty 2011). Further enhancement of grain yield must be achieved by increasing total biomass accumulation without increased N fertilizer inputs (Makino 2011). Improving photosynthesis performance is the most promising approach to meet this challenge (von Caemmerer and Evans 2010; Long et al. 2015; Makino 2021).

Ribulose-1,5-bisphosphate carboxylase-oxygenase (Rubisco) (EC 4.1.1.39) catalyzes CO_2_ fixation during photosynthesis and the production of 2-phosphoglycolate in the photorespiratory pathway, and the amount of Rubisco is considered a rate-limiting factor for carbon fixation under current atmospheric CO_2_ and O_2_ concentrations as well as saturating light (Makino et al. 1985). In our previous research, transgenic rice plants (*RBCS*-sense rice plants, line Sr-26-8) with approximately 30% overproducing Rubisco compared to the wild-type were produced by transforming *Rubisco small subunit2* (*OsRBCS2*, RAP-DB id; Os12g0274700) complementary DNA with a sense direction (Suzuki et al. 2007). The *RBCS*-sense rice plants indicated that grain yields were increased up to 28% compared to wild-type plants in the isolated experimental paddy field under sufficient N fertilization (Yoon et al. 2020). Furthermore, the N contents of the above-ground sections and Rubisco contents in the flag leaves of *RBCS*-sense plants were higher than those of the wild-type rice plants during ripening periods, which may be reasons for the higher yields (Yoon et al. 2020). However, like N fertilization, the light-reception efficiency, which is affected by canopy architecture, individual leaf photosynthesis, leaf area and leaf lifespan, is also one of the important factors determining the total dry matter productions and yields in the crops (Adachi et al. 2019; Inagaki et al. 2021). In rice plants, all the leaves from the flag leaf down to the third leaf are known to export photosynthates to the panicles during ripening periods (Yoshida 1981). The flag leaves have the highest photosynthesis per unit leaf area, but penultimate leaves are larger in size and have greater potential photosynthesis per leaf (Yoshida 1981). In addition, it is pointed out that crops with longer grain-filling periods are subsequently higher yields (Gregersen et al. 2013; Thomas and Ougham 2014; Kamal et al. 2019; Shin et al. 2020). Thus, some imprecise points were left in our previous research (Yoon et al. 2020), such as the contributions to the yield of photosynthesis of leaves below the flag leaves with a relatively unfavorable light-reception in the canopy, and maintenance duration of higher photosynthesis of *RBCS*-sense rice plants during the ripening period.

This research investigated the photosynthetic capacity of leaves existing during the ripening period and their light-reception by analyzing canopy architecture using the stratified cutting method to identify factors affecting photosynthesis that led to increased grain yields of *RBCS*-sense rice plants. It was found that N had already been preferentially distributed into the flag leaves of the *RBCS*-sense plants compared to those of the wild-type rice plants at the early ripening stage. The greater N distribution to the flag leaves appeared to improve the photosynthetic capacity by increasing in the Rubisco contents, enlarging the leaf area, and extending the lifespan of the flag leaves in the *RBCS*-sense rice plants. Here, the effects of these physiological and architectural changes in *RBCS*-sense rice plants on yield were discussed.

## Results

### Total Dry Matter Production and Brown Rice Yield of the *RBCS*-Sense Rice Plants

Table 1 indicates the total dry matter production of the above-ground sections, brown rice yields per unit land area, and yield components of the wild-type and *RBCS*-sense rice plants. In the plots under 15 g N m^−2^ fertilization, the total dry matter production and brown rice yield of *RBCS*-sense plants showed 10% and 16% increases compared to wild-type rice plants, respectively (*t*-test; *p* = 0.005 and *p* = 0.001, respectively) (Table 1). The weight of the single brown rice and the ratio of filled spikelets were 5% and 6% higher in *RBCS*-sense plants than in wild-type rice plants, respectively (*t*-test; *p* = 0.001 and *p* = 0.000, respectively) (Table 1). Total spikelet numbers were not significantly different between the two genotypes (*t*-test; *p* = 0.216) (Table 1). These results showed that the increased grain yield was caused by a higher ratio of filled spikelets and single weight of brown rice. In contrast, in cultivation plots applied with 8 g and 0 g N m^−2^ fertilizer, no increase in total dry matter production of *RBCS*-sense was observed, and the brown rice yield was significantly lower in *RBCS*-sense rice plants in the 0 g N m^−2^ plot (*t*-test; *p* > 0.05) (Table 1). Similar results were observed in our previous research (Yoon et al. 2020).

### High Photosynthetic Capacity in Flag Leaves of *RBCS*-Sense Rice Plants Throughout the Ripening Period Under Sufficient N Fertilization

**Fig. 1:**
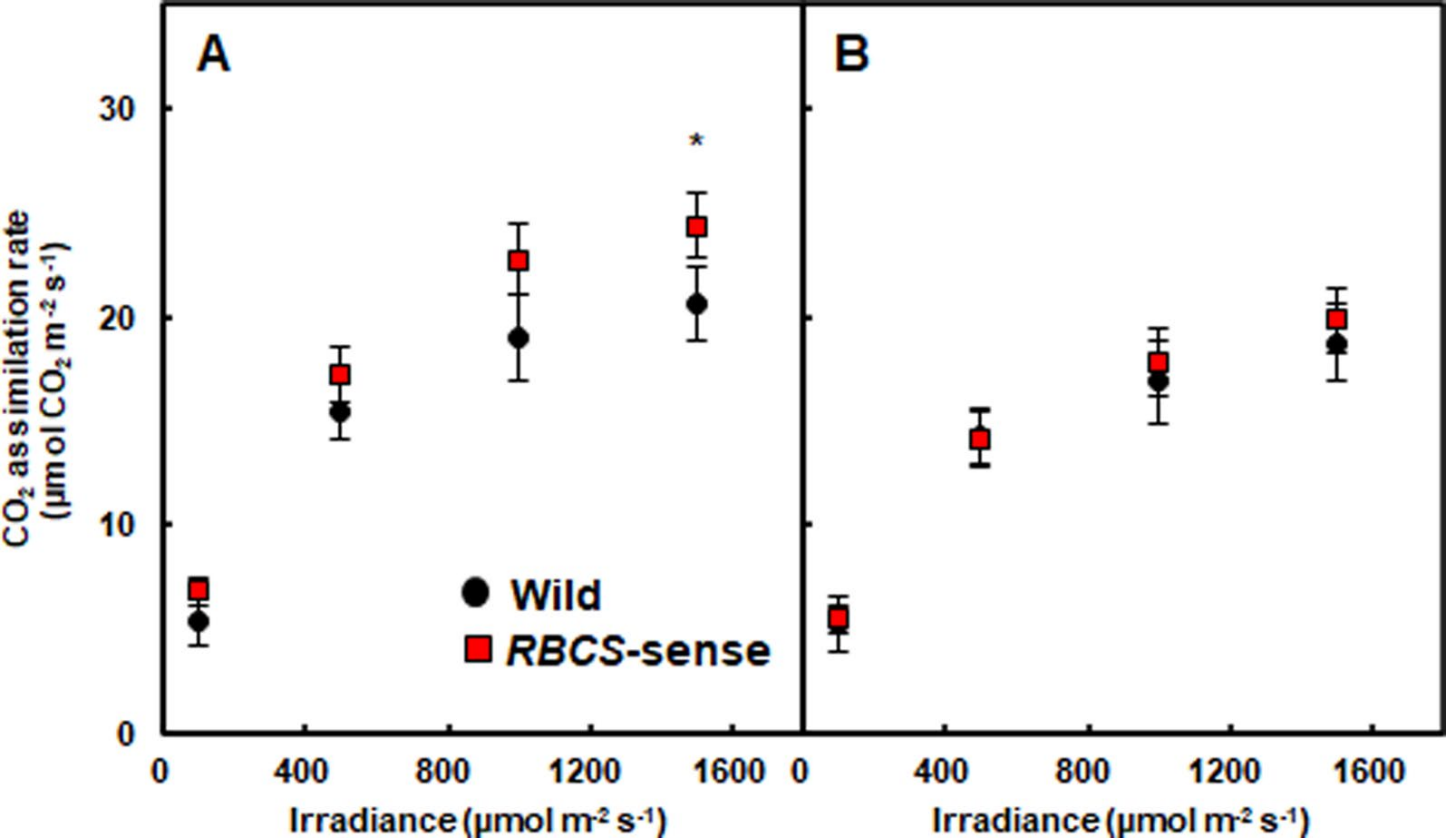
CO_2_ assimilation rate in the flag leaves (**A**) and penultimate leaf blades (**B**) of the wild-type and *RBCS*-sense rice plants at the early ripening stage in the plots applied with 15 g N m^−2^ fertilizer. The CO_2_ assimilation rates were measured at 1 DAH for flag leaves and 4 DAH for the penultimate leaves using LI-6400XT (Li-Cor). Mean values ± the standard error of 5–7 independent rice plants is shown. The wild-type and *RBCS*-sense rice plants are represented by black circles and red squares, respectively. *p < 0.05 between the wild-type and *RBCS*-sense rice plants using Student’s *t-*test. The abbreviations stand as following: “DAH”; days after heading, “*RBCS*-sense”; transgenic rice plants overproducing Rubisco, “Wild”, wild-type rice plant

In this research, the ripening period was divided into three stages and was defined as follows: early from the heading date to 17 days after heading (DAH), middle from 18 to 34 DAH, and late from 35 to the harvesting date. The CO_2_ assimilation rates per unit leaf area were measured in the flag leaves and penultimate leaves (leaves just below flag leaves) of wild-type and *RBCS*-sense rice plants at one and four DAH, respectively, in the early ripening stage (Fig. 1). The CO_2_ assimilation rates in flag leaves were approximately 18% higher in the *RBCS*-sense than in the wild-type rice plants under strong light conditions, at 1500 µmol quanta m^−2^ s^−1^ (*t*-test; *p* = 0.029) (Fig. 1A). Under other light conditions, such as 1000, 500, and 100 µmol quanta m^−2^ s^−1^, the CO_2_ assimilation rates of the *RBCS*-sense tended to be higher than those of wild-type plants, while there were no significant differences (*t*-test; *p* = 0.061 at 1000, *p* = 0.283 at 500, and *p* = 0.514 at 100 µmol quanta m^−2^ s^−1^, respectively) between the two genotypes (Fig. 1A). In contrast, there were no obvious differences in the CO_2_ assimilation rates of the penultimate leaves between wild-type and *RBCS*-sense rice plants (*t*-test; *p* > 0.05) (Fig. 1B).

**Fig. 2:**
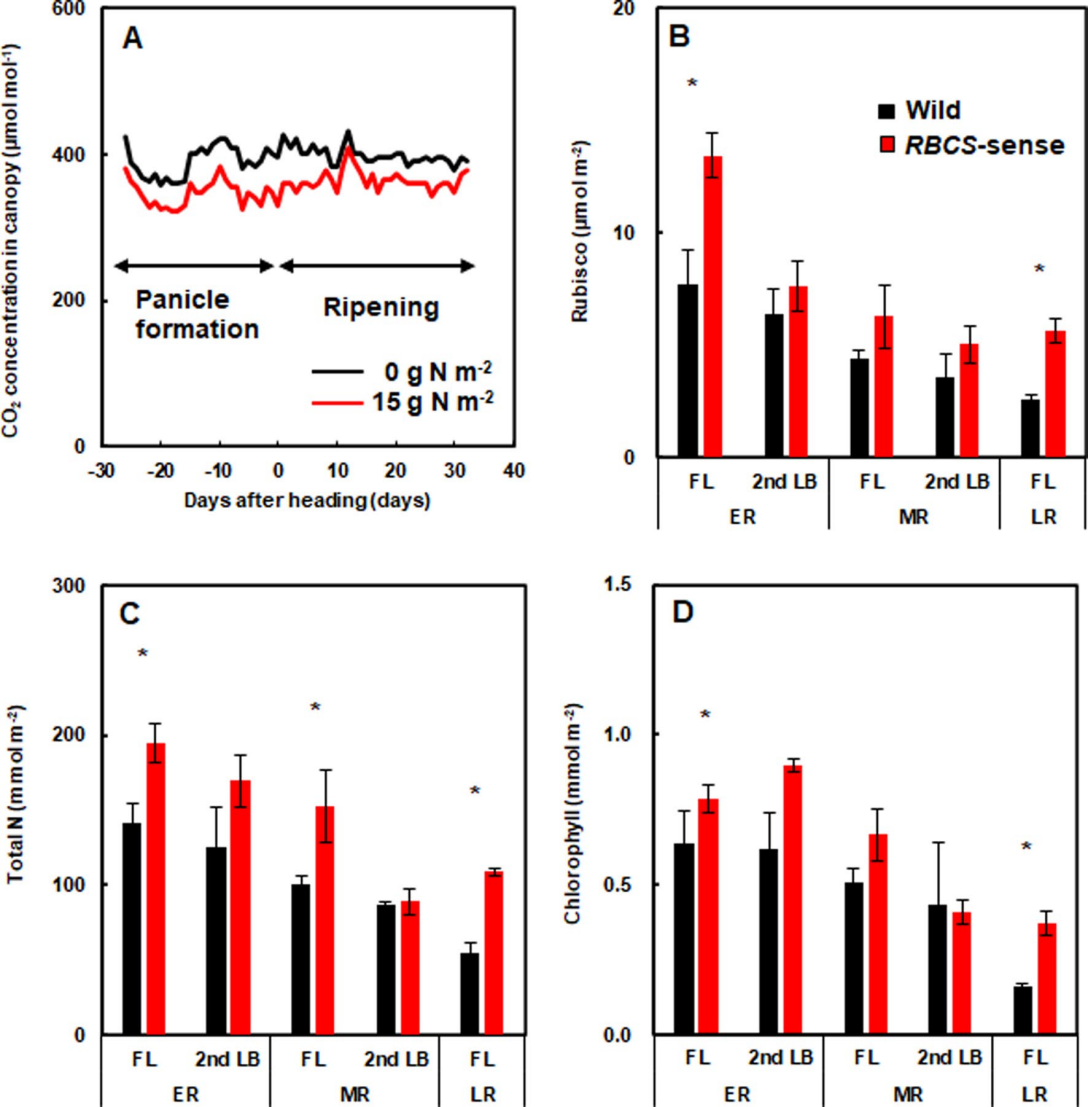
Changes in CO_2_ concentrations at airspace 10 cm below the top of the canopies of *RBCS*-sense rice plants throughout reproductive and ripening periods in the plots applied with 15 and 0 g N m^−2^ fertilizer (**A**). Comparisons of Rubisco (**B**), total N (**C**), and Chl (**D**) contents per unit land area of flag leaves between wild-type and *RBCS*-sense rice plants at the early, middle, and late stages of ripening period in the plots applied with 15 g N m^−2^ fertilizer. The CO_2_ concentrations in the canopies of the *RBCS*-sense rice plants were measured throughout − 26 to 35 DAH as reproductive and ripening periods (**A**). The flag and penultimate leaves of the wild-type and *RBCS*-sense rice plants for measuring Rubisco, total N and Chl contents were harvested from 4 to 8 DAH in early, from 26 to 30 DAH in the middle, and 47 DAH in the late ripening stages (**B**–**D**). The arrows represent the reproductive (panicle formation) and ripening periods. Mean values ± the standard error of 3–5 independent of the flag leaves and penultimate leaf blades of wild-type and *RBCS*-sense rice plants, respectively. *p < 0.05 between the wild-type and *RBCS*-sense rice plants using Student’s *t-*test. The abbreviations stand as follows: “Chl”; chlorophyll, “DAH”; days after heading, “ER”; early ripening stage, “FL”; flag leaves, “LR”; late ripening stage, “MR”; middle ripening stage, “PL”; penultimate leaf blade, “*RBCS*-sense”; transgenic rice plants overproducing Rubisco, “Wild”; wild-type rice plant

The atmospheric CO_2_ concentrations in the canopy of the *RBCS*-sense rice plants applied with 0 g and 15 g N m^−2^ N fertilizer were measured from –26 to 35 DAH as reproductive (panicle formation) and ripening periods. The atmospheric CO_2_ concentrations in the 15 g N m^−2^ plot were lower than in the 0 g N m^−2^ plot throughout the reproduction period, with a maximum depletion of 70 µmol mol^−1^, showing that photosynthesis was more active in the 15 g N m^−2^ than those in the 0 g N m^−2^ plot (Fig. 2A).

Total N, Rubisco, and chlorophyll (Chl) contents in the flag and penultimate leaves were measured from 4 to 8 DHA in the early, from 26 to 30 DAH in the middle, and 47 DAH in the late ripening stages. The amounts of Rubisco per unit land area in the flag leaves of the *RBCS*-sense rice plants were approximately 76–150% higher than those of the wild-type rice plants throughout the ripening period, although there were no significant differences in Rubisco contents between the two genotypes at the middle ripening period (*t*-test; *p* = 0.004 at the early ripening stages, *p* = 0.058 at the middle ripening stages, and *p* = 0.003 at the late ripening stages) (Fig. 2B). In the penultimate leaves, there were no differences in Rubisco contents at the early and middle ripening stages between the two genotypes (*t*-test; *p* > 0.05) (Fig. 2B). The total nitrogen and Chl contents also exhibited similar trends to the Rubisco contents in the flag leaves and penultimate leaves between the two genotypes (Fig. 2C, D). The total N contents in the flag leaves of *RBCS*-sense plants were 38–101% higher than those of wild-type rice plants (*t*-test; *p* = 0.023 at the early ripening stages, *p* = 0.049 at the middle ripening stages, and *p* = 0.002 at the late ripening stages), although those in penultimate leaves were almost the same (*t*-test; *p* > 0.05) at the early and middle ripening stages (Fig. 2C). The Chl contents in the flag leaves of *RBCS*-sense plants were 34%–103% higher than those of wild-type rice plants (*t*-test; *p* = 0.041 at the early ripening stages and *p* = 0.002 at the late ripening stages), although there were no significant differences at the middle ripening stage (*t*-test; *p* = 0.056). There were almost the same levels of Chl contents in the penultimate leaves between the two genotypes at the early and middle ripening stages (*t*-test; *p* > 0.05) (Fig. 2D). At the late ripening stage, the penultimate leaves were dead and in no measurable conditions (Fig. 2B–D).

### No Difference in Light Extinction in the Canopies Between Wild-Type and *RBCS*-Sense Rice Plants at the Early Ripening Stage

**Fig. 3:**
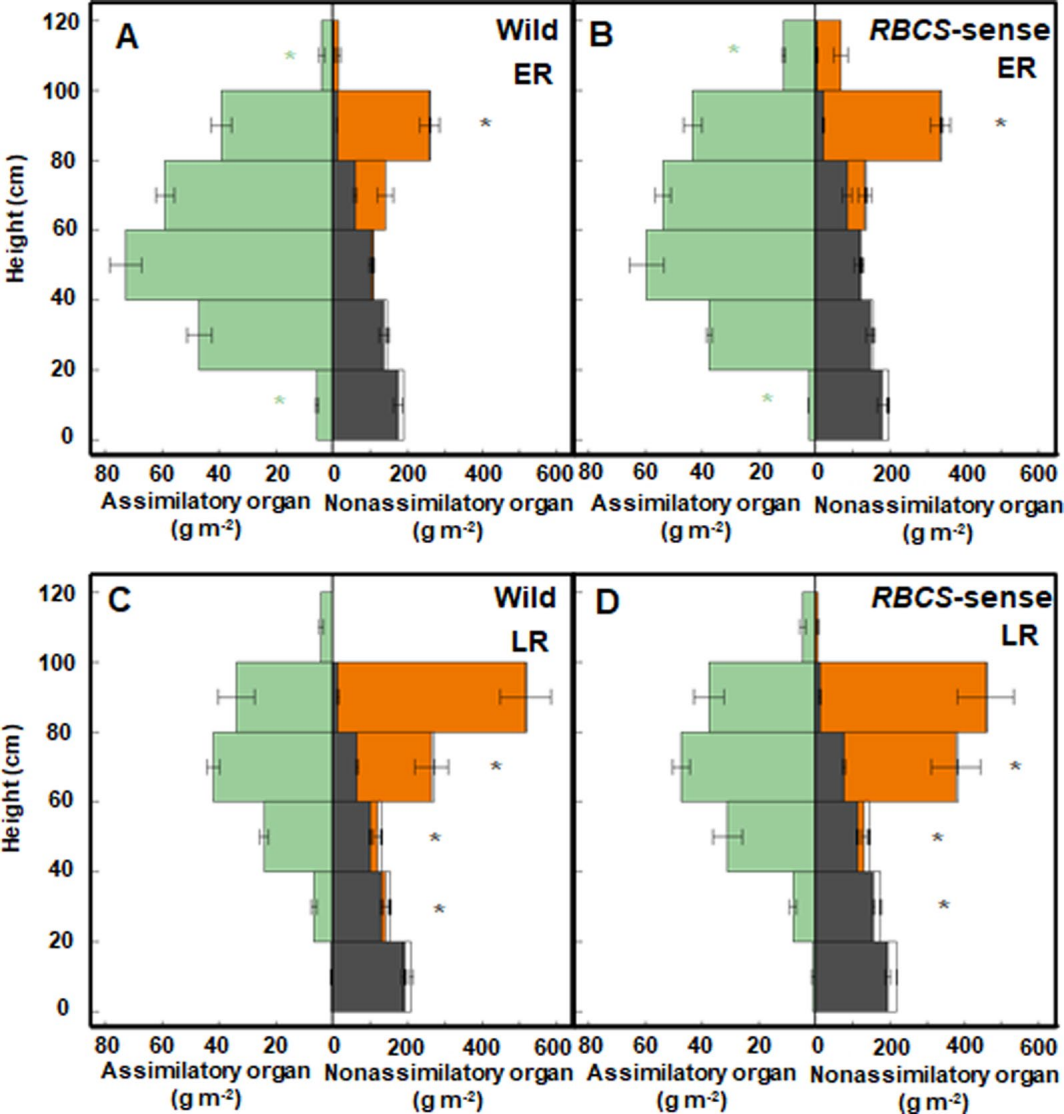
Changes in the wild-type (**A** and **C**) and *RBCS*-sense rice plants (**B** and **D**) canopy structures at 10 DAH in the early ripening stages and 49 DAH in the late ripening stages in the plots with 15 g N m^−2^ fertilizer. Mean values ± the standard error of three independent plots are indicated. *p < 0.05 between the wild-type and *RBCS*-sense rice plants using Student’s *t-*test. The dry weights of leaf blades, panicles, sheathes and stems, and dead organs are represented by green, orange, dark gray, and white bars, respectively. The abbreviations stand as follows: “DAH”; days after heading, “ER”; early ripening stage, “LR”; late ripening stage, “*RBCS*-sense”; transgenic rice plants overproducing Rubisco, “Wild”; wild-type rice plants

**Fig. 4:**
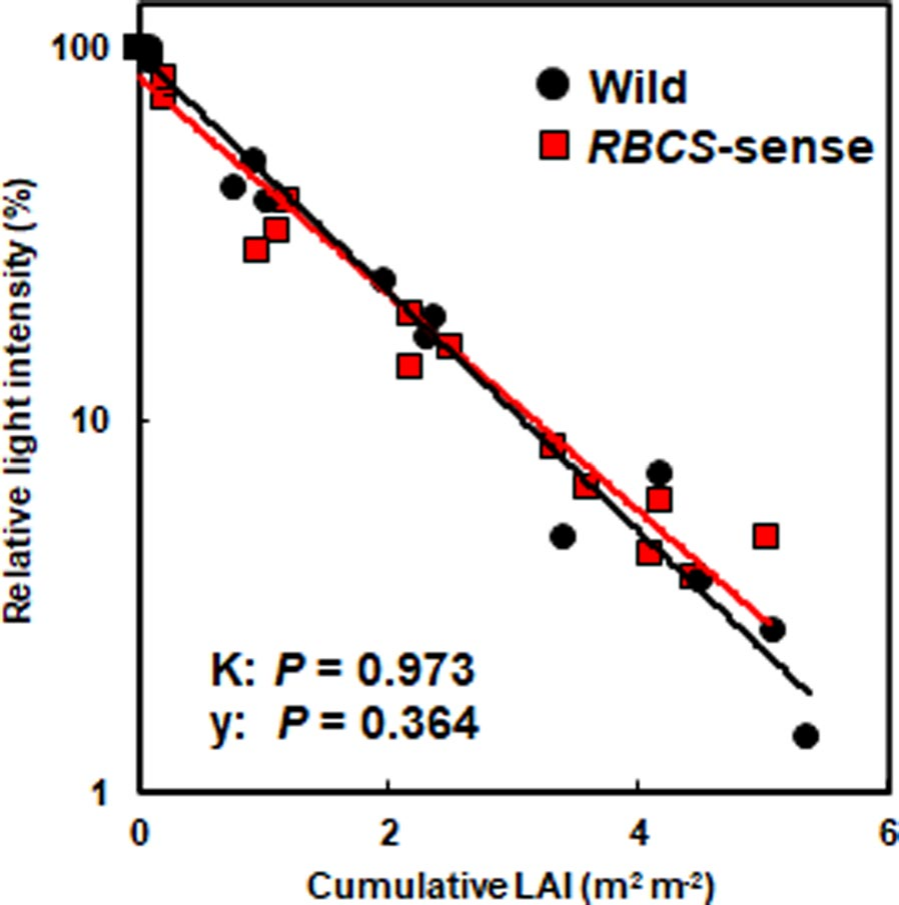
Relationships between relative light intensity and cumulative leaf area index (LAI) of the wild-type and *RBCS*-sense rice plants at 10 DAH in the early ripening stage in the plots applied with 15 g N m^−2^ fertilizer. The wild-type and *RBCS*-sense rice plants are represented by black circles and red squares, respectively. The slope of the line represents the light extinction coefficient (K). Covariance analyses were conducted between wild-type and *RBCS*-sense rice plants, and no significant differences were detected in the light extinction coefficient (K) and the intercept (y). The abbreviations stand as follows: “LAI”; leaf area index, “*RBCS*-sense”; transgenic rice plants overproducing Rubisco, “Wild”; wild-type rice plants

This current research showed that changes in total dry matter of above-ground sections (shoots) at 10 DAH in the early (Fig. 3A, B) and 49 DAH in late ripening stages (Fig. 3C, D) of the wild-type and *RBCS*-sense rice plants had similar trends to previous research; there were no differences (*t*-test; *p* = 0.135) in the total dry matter between the two genotypes at early ripening, but at the late ripening stage, there were significant differences (*t*-test; *p* = 0.001) (Yoon et al. 2020, Additional file 1: Figure S1). However, the stratified cutting analyses of this research found differences in canopy architecture, i.e., organ-specific dry weight along the vertical axis, between two genotypes from the early ripening stage. Three sets of samples comprised four neighboring hills with an average number of stems or panicles of wild-type and *RBCS*-sense rice plants at 10 DAH in the early ripening stages, and 49 DAH in the late ripening stages were selected from each 15 g N m^−2^ plot. The stem or panicle number per unit land area calculated from the four sampled hills is indicated in Additional file 2: Table S1. The dry weights of the leaf blades, known as the photosynthetic assimilatory organs, were 180% higher (*t*-test; *p* = 0.004) in the uppermost layer (100–120 cm) in *RBCS*-sense plants than in the wild-type rice plants at the early ripening stage, although there were no differences of those in 20–100 cm layer between the two genotypes (*t*-test; *p* > 0.05) (Fig. 3A, B). The dry weights of leaf blades in the bottom layer (0–20 cm), where senescent leaf blades were likely to die, were lower (*t*-test; *p* = 0.006) in *RBCS*-sense plants than in wild-type rice plants (Fig. 3A, B). The total dry weights of leaf blades in wild-type and *RBCS*-sense rice plants were not different (*t*-test; *p* = 0.876) (Additional file 1: Figure S1), and significant differences were observed only if stratified cutting was detected (Fig. 3A, B). From the results of relationships between relative light intensity and cumulative leaf area index (LAI) of wild-type and *RBCS*-sense rice plants, there were no differences (Spearman rank correlation, *p* = 0.000 in the wild-type and *RBCS*-sense rice plants, respectively; covariance analysis, K: slope *p* = 0.973, y-axis intercept *p* = 0.364) in the light extinction between canopies of the two genotypes (Fig. 4). These results were obtained due to the low ratio of the dry weights of leaf blades in the uppermost layer relative to those of total leaf blades, approximately 1.8% in the wild-type and approximately 5.4% in *RBCS*-sense rice plants. In contrast, the total dry weights of leaf blades of *RBCS*-sense plants were 16% higher (*t*-test; *p* = 0.011) than those of wild-type rice plants at the late ripening stage (Additional file 1: Fig. S1), although there were no significant differences (*t*-test; *p* > 0.05) in the dry weights of leaf blades at the same stage through the stratified distribution (Fig. 1C, D).

In the nonassimilatory organs, the stratified distribution of the dry weights of leaf sheaths and stems in the 80–100 cm and 20–80 cm layers was higher (*t*-test; *p* = 0.013 in the 80–100 cm layer at early, and *p* = 0.048 in the 60–80 cm, *p* = 0.011 in the 40–60 cm, *p* = 0.002 in the 20–40 cm layers at the late ripening stages), in *RBCS*-sense plants than in wild-type rice plants at early (Fig. 3A, B) and late ripening stages (Fig. 3C, D), respectively. The total dry weights of the leaf sheaths and stems were greater in the *RBCS*-sense plants at the late ripening stage (*t*-test; *p* = 0.009) than in the wild-type rice plants (Additional file 1: Fig. S1), although they were not different at the early ripening stage (*t*-test; *p* = 0.334) (Additional file 1: Fig. S1). In the panicles, there were no significant differences in dry weights in the individual layer between the two genotypes at either stage (*t*-test; *p* > 0.05) (Fig. 3). The total dry weights of the panicles of *RBCS*-sense plants were 5% higher (*t*-test; *p* = 0.011) than those of wild-type rice plants (Additional file 1: Fig. S1), supporting the results of the increased brown rice yields in *RBCS*-sense plants compared to wild-type rice plants (Table 1). The dry weights of dead organs between the two genotypes were not different from each other (*t*-test; *p* > 0.05) at the early and late ripening stages and were very low compared with the total dry weight production (Fig. 3, Additional file 1: Fig. S1).

### Enlarged Leaf Area of the Flag Leaves in *RBCS*-Sense Rice Plants Grown Under Sufficient N Fertilization

The plant height was slightly higher (*t*-test; *p* = 0.026) in *RBCS*-sense plants than in wild-type rice plants (Table 2). The leaf area and length of flag leaves were approximately 9% and 13% larger (*t*-test; *p* = 0.028 and *p* = 0.001, respectively) in *RBCS*-sense plants than in wild-type rice plants, respectively (Table 2). In contrast, there were no significant differences in the area and length of penultimate leaves, the height of the lamina joints of flag leaves and penultimate leaves, between the two genotypes (*t*-test; *p* > 0.05) (Table 2). Thus, the higher plant heights of *RBCS*-sense rice plants were mainly due to the enlarged length of the flag leaves. Additionally, the flag leaves of *RBCS*-sense rice plants exclusively occupied the uppermost layer (100–120 cm), since the penultimate leaves of the two genotypes did not attain the uppermost layer because of the relationship between the lengths of penultimate leaves and the heights of their lamina joints (Fig. 3, Table 2).

**Table 1:** Comparisons of total dry matter, yield, and yield components in 2021

N fertilization (g N m^−2^)	Line	Total dry matter (m^−2^)	Brown rice yield (g m^−2^)	Total spikelets number (× 10^3^ m^−2^)	Single weight of brown rice (mg)	Ratio of filled spikelets (%)
15	Wild	1652 ± 31 (100)	630 ± 20 (100)	29.2 ± 0.8 (100)	25.7 ± 0.1 (100)	83.8 ± 1.0 (100)
	*RBCS*-sense	1811 ± 42 (109)^*^	733 ± 20 (116)^*^	30.6 ± 0.8 (104)	26.9 ± 0.1 (105)^*^	88.9 ± 0.7 (106)^*^
8	Wild	1369 ± 48 (100)	541 ± 19 (100)	23.2 ± 0.8 (100)	26.5 ± 0.1 (100)	88.0 ± 1.8 (100)
	*RBCS*-sense	1361 ± 35 (99)	509 ± 17 (94)	21.8 ± 0.5 (93)	26.5 ± 0.3 (100)	88.3 ± 1.4 (100)
0	Wild	706 ± 29 (100)	287 ± 13 (100)	11.7 ± 0.4 (100)	27.6 ± 0.2 (100)	88.5 ± 1.8 (100)
	*RBCS*-sense	630 ± 26 (89)	237 ± 12 (82)*	10.0 ± 0.4 (85)^*^	26.2 ± 0.2 (94)*	89.5 ± 1.6 (101)

Mean values ± the standard error of 15 independent plants is indicated. Statistical analysis was conducted using Student’s *t*-test (*p* < 0.05). Asterisk denotes a statistically significant difference in the total dry matter production of the above-ground section, brown rice yield, total spikelet number per unit land area, single grain weight of brown rice, and the ratio of filled spikelets between the wild-type and *RBCS*-sense rice plants. Values in parentheses indicate the percentage of the value of the wild-type rice plants. The abbreviations stand as follows: “*RBCS*-sense”; transgenic rice plants overproducing Rubisco, “Wild”; wild-type rice plant.

**Table 2:** Plant height, leaf area, leaf length, height of the lamina joints of the flag, and penultimate leaf blades on 10 DHA in the early ripening stage in the plots applied with 15 g N m^−2^ fertilizer

Line	Plant height (cm)	Leaf area (cm^2^)		Leaf length (cm)		Height of the lamina joints (cm)	
		FL	PL	FL	PL	FL	PL
Wild	115.8 ± 1.1 (100)	31.4 ± 0.9 (100)	34.1 ± 0.9 (100)	29.9 ± 0.9 (100)	36.2 ± 1.1 (100)	88.4 ± 1.1 (100)	61.0 ± 1.1 (100)
*RBCS*-sense	118.9 ± 0.5 (102)*	34.3 ± 0.9 (109)*	35.9 ± 0.9 (105)	33.9 ± 0.9 (113)*	34.9 ± 1.1 (96)	88.2 ± 4.7 (99)	61.9 ± 0.9 (101)

Data represent means ± the standard error (plant height: n = 26 in the wild-type and n = 20 in the *RBCS*-sense, leaf area of flag leaves: n = 60 in the wild-type and *RBCS*-sense, leaf area of the penultimate leaf blades: n = 51 in the wild-type and n = 49 in *RBCS*-sense, length of flag leaf: n = 35 in the wild-type and n = 34 in the *RBCS*-sense, leaf length of the penultimate leaf blades: n = 20 in the wild-type and n = 23 in the *RBCS*-sense, height of lamina joints: n = 30 in the wild-type and the *RBCS*-sense). *p < 0.05 between the wild-type and *RBCS*-sense rice plants using Student’s *t-*test. Values in parentheses indicate the percentage of the values of wild-type rice plants. The abbreviations stand as follows: “DAH”; days after heading, “FL”; flag leaves, “PL”; penultimate leaf blades, “*RBCS*-sense”; transgenic rice plants overproducing Rubisco, “Wild,”; wild-type rice plants

### Preferential N Partitioning to Flag Leaves in *RBCS*-Sense Rice Plants

**Fig. 5:**
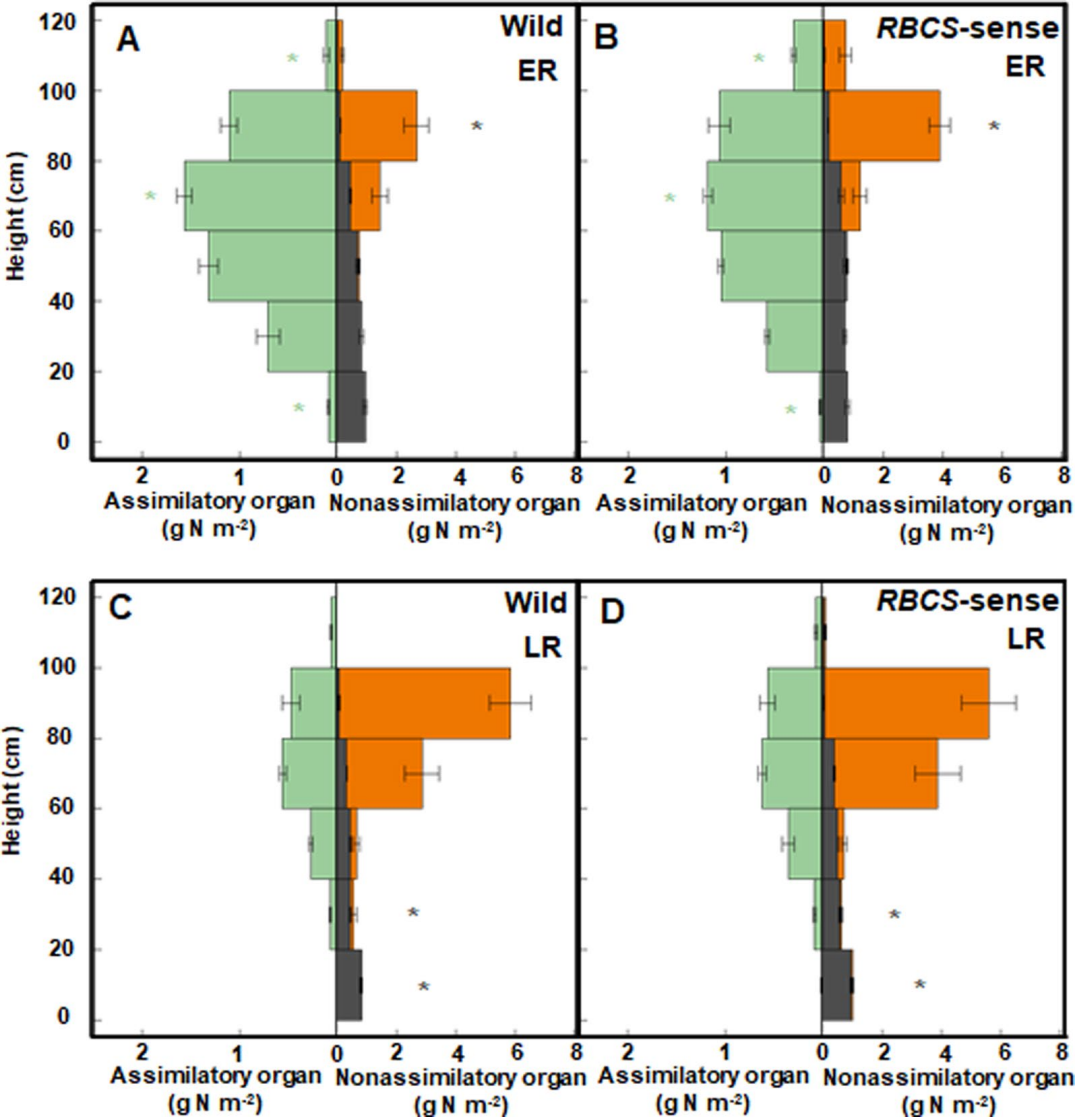
Changes in the vertical distribution of N content in the canopies of the wild-type (**A** and **C**) and *RBCS*-sense rice plants (**B** and **D**) at 10 DAH in early and 49 DAH in late ripening stages in plots applied with 15 g N m^−2^ fertilizer. Mean values ± the standard error of three independent plots are indicated. *p < 0.05 between the wild-type and *RBCS*-sense rice plants using Student’s *t-*test. The N contents of leaf blades, panicles, and sheathes and stems are represented by green, orange, and dark gray bars, respectively. The abbreviations stand as following: “ER”; early ripening stage, “LR”; late ripening stage, “N”; nitrogen, “*RBCS*-sense”; transgenic rice plants overproducing Rubisco, “Wild”; wild-type rice plants

There were no differences (*t*-test; *p* = 0.556) in the total N contents of above-ground sections between two genotypes at 10 DAH in the early ripening stage, although those of *RBCS*-sense plants were higher (*t*-test; *p* = 0.001) than those of wild-type rice plants at 49 DAH in the late ripening stage (Additional file 3: Fig. S2). However, the stratified cutting analyses showed differences in organ-specific N contents along the vertical axis between the two genotypes. Figure 5 indicates the vertical distribution of N in the canopies of wild-type and *RBCS*-sense rice plants at the early (Fig. 5A, B) and late ripening stages (Fig. 5C, D), respectively. The N contents of the leaf blades were 188% higher (*t*-test; *p* = 0.008) in the uppermost layer (100–120 cm) where flag leaves were found in *RBCS*-sense plants than in wild-type rice plants at the early ripening stage (Fig. 5A, B). In contrast, in the lower layer, 60–80 cm and 0–20 cm, where senescent leaf blades were present, the N contents of leaf blades were 25% and 57% lower (*t*-test; *p* = 0.012 and *p* = 0.026, respectively) in *RBCS*-sense plants than in wild-type rice plants, respectively, at the same stage (Fig. 5A, B). In the nonassimilatory organs, the N contents of the leaf sheaths and stems of *RBCS*-sense plants were higher (*t*-test; *p* = 0.027) than those of wild-type rice plants in the 80–100 cm layer at the early ripening stage, although there were no differences (*t*-test; *p* > 0.05) in those panicles between the two genotypes (Fig. 5A, B). In contrast, the total N contents of each organ in the wild-type and *RBCS*-sense rice plants were not different (*t*-test; *p* > 0.05) at the early ripening stage (Additional file 3: Fig. S2A).

In the late ripening stages, the total N contents of leaf blades, leaf sheaths and stems, and panicles in *RBCS*-sense rice plants were higher (*t*-test; *p* = 0.019 at leaf blades, *p* = 0.001 at leaf sheaths and stems, and *p* = 0.011 at panicles) than those in the wild-type rice plants at the late ripening stage (Additional file 3: Fig. S2B). The stratified distribution of the N contents of leaf sheaths and stems in 0–40 cm layers were higher (*t*-test; *p* = 0.019 in the 0–20 cm and *p* = 0.022 in the 20–40 cm layers) in *RBCS*-sense plants than wild-type rice plants, although there were no differences (*t*-test; *p* > 0.05) in N contents of leaf blades and panicles at the late ripening stage (Fig. 5C, D).

### Extended Lifespan of Flag Leaves in *RBCS*-Sense Rice Plants

**Fig. 6:**
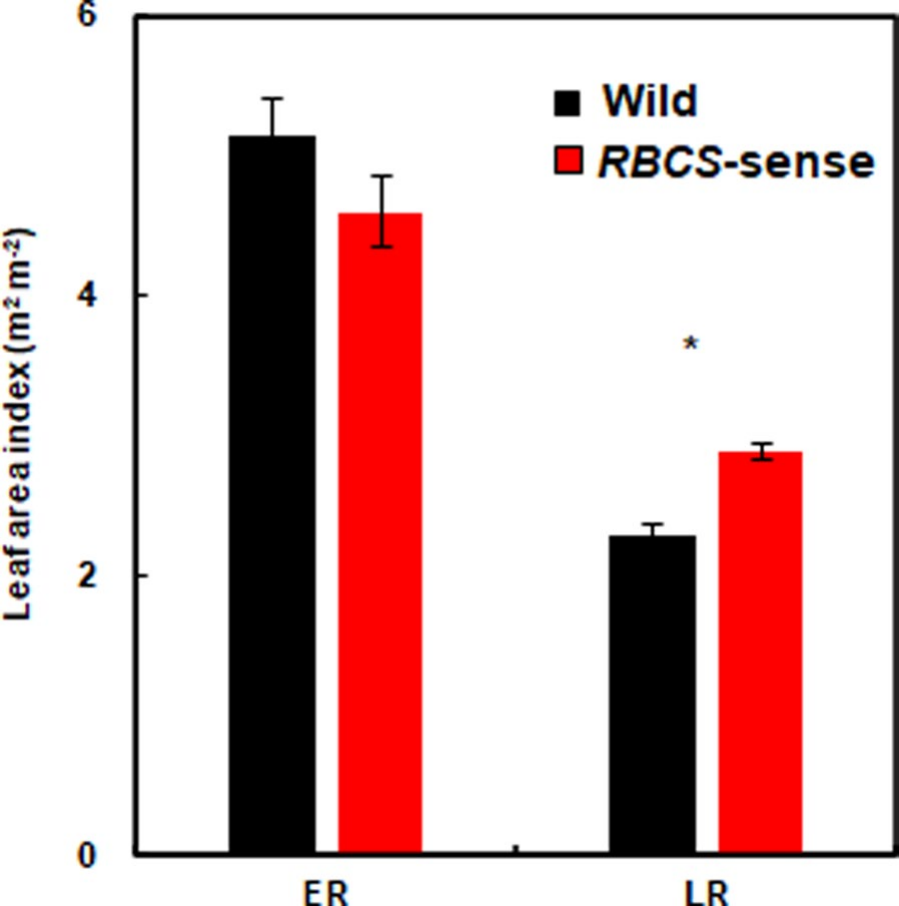
Changes in the leaf area index between 10 DAH in the early ripening stages and 49 DAH in the late ripening stages in the plots with 15 g N m^−2^ fertilizer. Mean values ± the standard error of three independent plots are indicated. *p < 0.05 between the wild-type and *RBCS*-sense rice plants using Student’s *t-*test. The wild-type and *RBCS*-sense rice plants are indicated by black and red bars, respectively. The abbreviations stand as follows: “ER”; early ripening stage, “LR”; late ripening stage, “*RBCS*-sense”; transgenic rice plants overproducing Rubisco, “Wild”; wild-type rice plants

The CO_2_ assimilation rate declines due to the decrease in total N, Rubisco, and Chl contents in leaves during leaf aging (Makino et al. 1985). Thus, the CO_2_ assimilation rate decline process and a decrease in LAI can be defined as leaf senescence. All the leaves of rice plants are senescent during the ripening period. The extended lifespan of leaves has a considerable effect on the yield of rice plants. In the current research, there were no significant differences in the LAI between the two genotypes at the early ripening stage, but the LAI was 26% higher (*t*-test; *p* = 0.003) in *RBCS*-sense plants than in wild-type rice plants at the late ripening stage (Fig. 6). These results indicated that the reduction rate in the LAI of the *RBCS*-sense plants during the ripening period was lower than that of the wild-type rice plants. Additionally, the Rubisco, total N, and Chl in the flag leaves of the *RBCS*-sense plants were higher than those in wild-type rice plants throughout the ripening period (Fig. 2B–D). These results indicate that the photosynthetic capacity was maintained at higher levels in the *RBCS*-sense plants than in wild-type rice plants throughout the ripening period.

### High Accumulation of Starch in the Leaf Sheaths and Stems of *RBCS*-Sense Rice Plants at the Late Ripening Stage

**Fig. 7:**
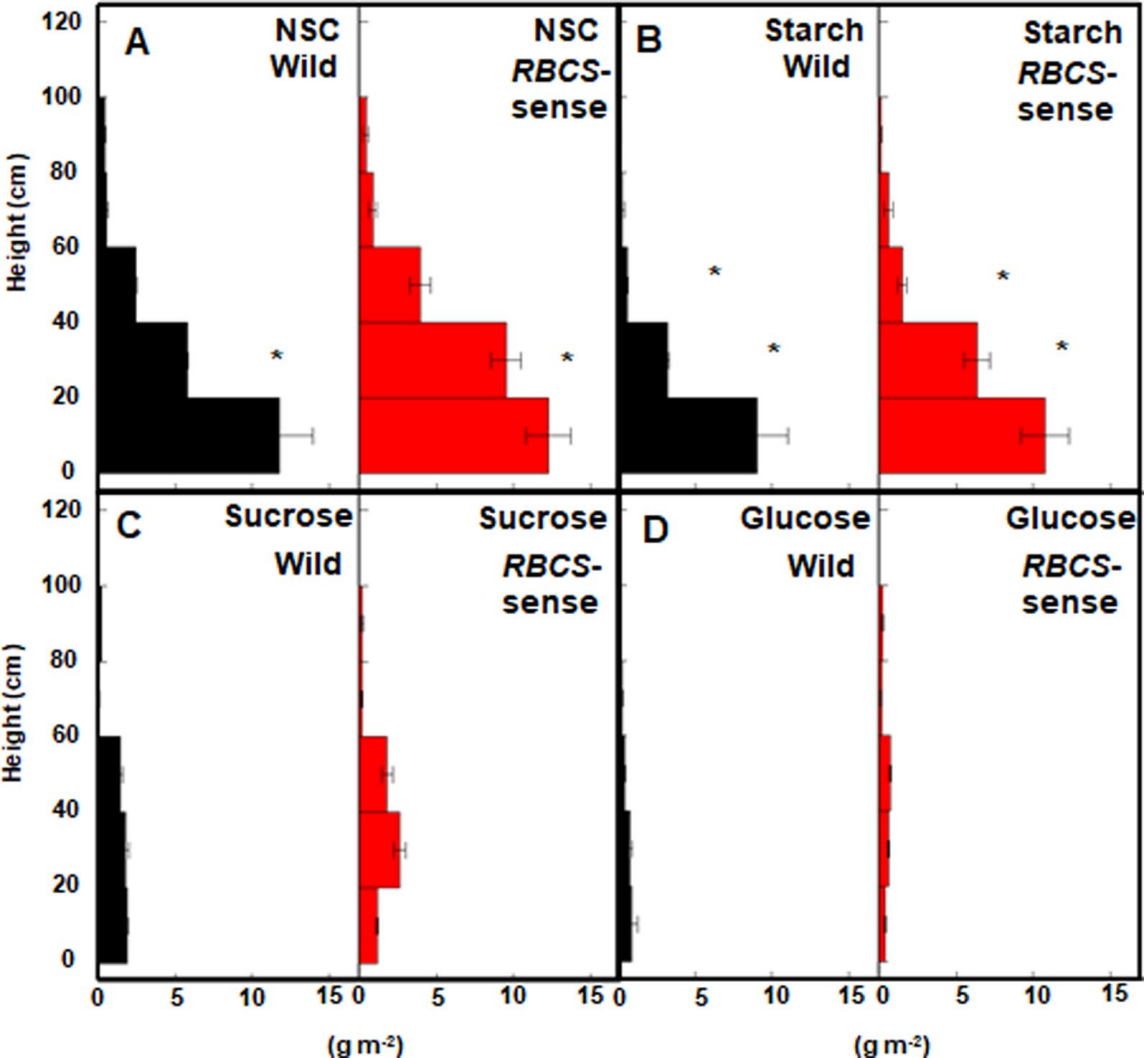
The vertical distribution of the contents of nonstructural carbohydrates (NSCs) in the leaf sheaths and stems of wild-type and *RBCS*-sense rice plants in the canopies at 49 DAH in the late-ripening stage in the plots with 15 g N m^−2^ fertilizer. The contents of NSCs (**A**) starch (**B**), sucrose (**C**), and glucose (**D**) are indicated. Mean values ± the standard error of three independent plots are shown. *p < 0.05 between the wild-type and *RBCS*-sense rice plants using Student’s *t-*test. The wild-type and *RBCS*-sense rice plants are represented by black and red bars, respectively. The abbreviations stand as follows: NSC; nonstructural carbohydrates, “*RBCS*-sense”; transgenic rice plants overproducing Rubisco, “Wild”; wild-type rice plants

To evaluate the photosynthetic capacity of rice plants during ripening periods, it is important to measure the amounts of photosynthates accumulated in the sheaths and stems at the harvest stage. When the nonstructural carbohydrate (NSC) contents in the sheathe and stems used for stratified cutting were measured, those of *RBCS*-sense plants in the 20–40 cm layer were approximately 29% higher (*t*-test; *p* = 0.019) than those of wild-type rice plants (Fig. 7A). Looking at the components of NSCs, the starch contents of the sheaths and stems in *RBCS*-sense plants in the 20–60 cm layers were significantly approximately 107% higher (*t*-test; *p* = 0.020 in the 20–40 cm layer and *p* = 0.040 in the 40–60 cm layer) than in wild-type rice plants (Fig. 7B). In contrast, there were no clear differences (*t*-test; *p* > 0.05) in the contents of sucrose and glucose in each layer (Fig. 7C, D).

## Discussion

Over the past decade, many crops with enhanced photosynthetic capacity have been developed to improve yields (Bailey-Serres et al. 2019). However, at the field level, there were no convincing examples of increased yields due to photosynthesis improvement until 2019 (Sinclair et al. 2019). For this reason, there has been skepticism about whether the improvement of photosynthetic capacity could increase crop yields (Sinclair et al. 2019). Long (2020) evaluated our previous research (Yoon et al. 2020) using *RBCS*-sense rice plants in paddy fields as having provided the clearest evidence that this skepticism may be misplaced. Here we conclude that important factors of the increased grain yields of the *RBCS*-sense rice plants were improvements of the photosynthetic capacity by physiological and architectural changes in the flag leaves due to higher leaf N and Rubisco contents throughout the ripening periods under sufficient N fertilization.

### Flag Leaves Largely contribute to the Increased Yield of *RBCS*-Sense Rice Plants

During the ripening periods, the *RBCS*-sense rice plants exhibited increased N absorption, and high levels of Rubisco protein in the flag leaves were maintained throughout the ripening periods (Yoon et al. 2020). In addition to the relationship between the two phenomena, other factors that should have been clarified relating to the increased yields of the *RBCS*-sense rice plants, such as the effect of the light-reception postures in the canopies and of the leaves below the flag leaves existing in the ripening stage on the yields, remained unsolved. Our current results clearly showed that N was preferentially distributed to the flag leaves of the *RBCS*-sense rice plants at the early ripening stage (Figs. 2C, 5A, B). The greater N distributions to the flag leaves contributed a greater increase in Rubisco contents with enhanced photosynthetic capacity in the flag leaves under sufficient N fertilization (Figs. 1, 2). Additionally, the flag leaves of the *RBCS*-sense plants had an enlarged leaf area compared to wild-type plants, although the leaf area did not vary in the penultimate leaves (Table 2). While the enlarged flag leaves of the *RBCS*-sense plants occupied larger spatial areas of the uppermost layers in the canopies at the early ripening stage compared to wild-type rice plants (Fig. 3A, B), it did not substantially prevent the penetration of light into the lower leaves (Fig. 4). Thus, the light-receptions in the canopies of wild-type and *RBCS*-sense rice plants are not different. The photosynthetic activity of the penultimate leaves between two genotypes was almost the same level at the early ripening stage (Fig. 1B). For these reasons, it is considered that the leaves in layers below flag leaves of the *RBCS*-sense rice plants did not contribute to the increased yields. The flag leaf in rice plants is the last leaf to appear and expands fully approximately 10 days before heading (Hoshikawa 1989; Mae and Ohira 1981). In rice plants, photosynthesis in flag leaves is the greatest contributor to ripening (Hoshikawa 1989; Yoshida 1981; Cook et al. 1983). Many physiological and molecular biological researches have indicated that flag leaves have the highest photosynthetic activity in the crop canopy after heading, which is closely correlated with grain yield (Yoshida 1981; Adachi et al. 2017, 2019; Gu et al. 2012; Takai et al. 2013; Carmo-Silva et al. 2017; Honda et al. 2021). Thus, it was concluded that the increases in the yields of the *RBCS*-sense plants compared to the wild-type rice plants are largely due to the functions of enlarged flag leaves with high photosynthetic capacity under sufficient N fertilization.

The sizes of the flag leaves are thought to be determined by the N contents of the rice plants during their emergence (Fukushima 2007). However, in previous study, no differences were found in the total N contents in above-ground sections between wild-type plants and *RBCS*-sense rice plants until heading (Yoon et al. 2020). Thus, it is difficult to explain merely the mechanisms of flag leaves enlargement in the *RBCS*-sense rice plants (Table 2). A possible explanation follows. With aging of rice leaves, Rubisco content drops faster than Chl content in rice leaves (Makino et al. 1983). N compounds derived from the degradation of Rubisco are transported to newly formed organs such as young leaves that require more N for their rapid growth (Mae et al. 1984; Ishida et al. 2014; Wada et al. 2015). Compared to wild-type plants, individual leaves of *RBCS*-sense rice plants distributed more amounts of N to Rubisco than Chl contents (Sudo et al. 2014). The increase amounts of Rubisco in full-expansion leaves of the *RBCS*-sense plants, compared to those of the wild-type rice plants, rapidly disappeared with aging (Suzuki et al. 2009). These facts suggest that more amounts of N from Rubisco degradations in senescent leaves below flag leaves occurred in the *RBCS*-sense plants compared to the wild-type rice plants. The larger N in the senescent leaves of the *RBCS*-sense rice plants might be transferred to the flag leaves which were rapidly growth at emergence, resulting in the enlargement of the flag leaves in the *RBCS*-sense rice plants.

### Low CO_2_ Conditions May Promote N Uptake of the *RBCS*-Sense Rice Plants

Our previous research using artificial growth chambers showed that growth of *RBCS*-sense plants was further promoted at low CO_2_ concentration compared to the wild-type rice plants, and the growth promotion may be caused by increasing the total leaf N content with increasing N uptake (Sudo et al. 2014). This study observed a CO_2_ depletion of up to 70 μmol mol^−1^ from atmospheric levels in the 15 g N m^−2^ plots compared to the 0 g N m^−2^ plots cultivated with the *RBCS*-sense rice plants during the ripening period (Fig. 2A). The *RBCS*-sense rice plants showed increased N uptake during the ripening periods in the paddy fields (Yoon et al. 2020). This CO_2_ depletion may have facilitated the N uptake of the *RBCS*-sense rice plants in sufficient N compared to those that were insufficient N fertilization during ripening periods.

### Increasing the Amount of N Distributed to Flag Leaves in *RBCS*-Sense Rice Plants Further Increases the Amount of Rubisco and Prolongs Leaf Lifespan

The greater N distribution to the flag leaves of the *RBCS*-sense rice plants gives two additional effects: (1) further increase in Rubisco content per unit leaf area (Fig. 2B, C), and (2) extending the lifespan of the flag leaves (Fig. 6). Rubisco increases in amounts and production efficiency as N increases the leaf blades (Makino et al. 1994). Therefore, the overproduction of Rubisco in the flag leaves of *RBCS*-sense rice plants due to greater N distribution to the flag leaves may have led to higher photosynthetic capacity. To increase yields in crop plants including rice, it is essential to enhance the CO_2_ assimilation rates and to maintain higher photosynthesis throughout the ripening period (Yoshida 1981; Parry et al. 2011; Lee and Tollenaar 2007; Araus et al. 2021; Makino 2021; Paul 2021). Our results indicated that the LAI duration of *RBCS*-sense rice plants was longer than that of wild-type plants (Fig. 7), and Rubisco, total N, and Chl contents in the flag leaves remained higher in *RBCS*-sense plants until the late ripening period (Fig. 2B–D). This means that *RBCS*-sense rice plants have a long-term high photosynthetic capacity to manufacture greater photosynthates throughout the ripening period under sufficient N fertilization. Unlike sufficient N fertilization, yields of *RBCS*-sense plants did not increase in plots with insufficient N fertilization of 0 or 8 g N m^−2^ (Table 1, Yoon et al. 2020). These results suggested that Rubisco overproduction is not always effective for increasing yields. Since N reallocation to Rubisco from other key components limiting photosynthesis occurs in *RBCS*-sense rice plants (Suzuki et al. 2007), we think that N deficiency tends to be slightly promoted under insufficient N conditions.

### Greater Source Capacity as High Photosynthetic Activity in the *RBCS*-Sense Rice Plants

*RBCS*-sense rice plants showed approximately twofold increased starch accumulation in the leaf sheaths and stems in lower layers than wild-type rice plants (Fig. 7B). In rice plants, photosynthates accumulate in spikelets mostly during the early and middle periods of ripening (Yoshida 1981). In the later ripening period, excess photosynthates that are no longer stored in the spikelets accumulate in the leaf sheaths and stems (Cock and Yoshida 1972; Nagata et al. 2002). As indicated in Table 1, the ratio of filled spikelets in the *RBCS-*sense rice plants in the 15 g N m^−2^ plots was 88.9%, close to the upper limit of the ratio, while that in the wild-type rice plants was 83.8%, which still had some space to accumulate dry matter in their spikelets. Differences in the ratio of filled spikelets and the amount of starch accumulated in the leaf sheath and stem between the two genotypes could mainly be attributed to the difference in their photosynthetic abilities of flag leaves during the ripening period. These facts indicated that the *RBCS*-sense rice plant has greater source capacity and high photosynthesis than its sink size. In our previous research, high-yielding *japonica* rice cultivars with large-grain; Akita 63 exhibited high grain yields but a lower ratio of filled spikelets under sufficient N fertilization in paddy fields (Mae et al. 2006; Makino et al. 2020). This phenomenon seems to be due to the limited photosynthetic capacity relative to its sink size caused by its spikelet size of Akita 63. If rice plants with large sink sizes such as Akita 63 are crossed with *RBCS*-sense rice plants with greater source capacity, further increases in grain yields can be expected.

## Conclusion

In our previous research, transgenic rice plants overproducing Rubisco (*RBCS*-sense rice plants) showed increased grain yields when cultivated in an experimental paddy field under sufficient N fertilization (Yoon et al. 2020). This research clearly showed that *RBCS*-sense rice plants have enlarged flag leaves with high photosynthetic capacity due to the higher Rubisco content and long lifespan throughout the ripening period. In addition, there was no difference in the light-reception of leaves positioned at the lower layers in the canopy between the wild-type plants and *RBCS*-sense rice plants. However, photosynthetic capacity of penultimate leaves between the two genotypes was the same, suggesting that the lower leaves did not contribute to increase in yields of *RBCS*-sense rice plants. These results indicated that the greater N distributions to the flag leaves led to improvement of photosynthetic capacity due to the increase in Rubisco contents, enlargement of the leaf area and extension of the lifespan of flag leaves, resulting in the increased yields of the *RBCS*-sense rice plants.

## Methods

### Plant Material and Field Growth Conditions

Rice (*Oryza* sativa L. cv. Notohikari) was transformed with *OsRBCS2* (gene identifier Os12g0274700 in The Rice Annotation Project Database; https://rapdb.dna.affrc.go.jp/) cDNA in the sense orientation under the control of its own *OsRBCS2* promoter, and transgenic lines with a substantially increased Rubisco content were selected (*RBCS*-sense rice plants, line name; Sr-26-8) (Suzuki et al. 2007). T3 progenies of line Sr-26-8 were backcrossed to non-transformed (wild-type) rice plants (*Oryza* sativa L. cv. Notohikari) twice (BC1 and BC2), and BC2F1 seeds were obtained by selfing BC2. From BC2F2 seeds obtained by selfing BC2F1, plants with homozygous for the *OsRBCS2* transgene were selected. In the present research, BC2F6 progeny of line Sr-26-8 expressing approximately 130% of the Rubisco of wild-type plants were used. The wild-type rice plants were used as controls.

Rice plants of two genotypes were grown in the Isolated Farm for Genetically Modified Plants (Isolated Paddy Field) (Hossain et al. 2000; Yoon et al. 2020) of the Field Science Center, Tohoku University (Kawatabi Field Center; 38°44′ N, 140°45′ E, at 140 m altitude) in 2021. The soil in the isolated paddy field (Furukawa sandy alluvial soil) was sand, had pH5.8, 12.0 g total C kg^−1^, 1.0 g total N kg^−1^ and 10.0 cmol ( +) kg^−1^ cation exchange capacity (Yoon et al. 2020). Seedlings were grown in an isolated greenhouse beginning on 18th April in 2021. Seedlings were planted in the paddy field at a hill spacing of 0.300 × 0.163 m (20.5 hills m^−2^) with three seedlings per hill. In 2021, the sizes of the respective plots for wild-type and *RBCS*-sense rice plants were 13.3 m^2^ (3.6 m wide and 3.7 m long) and 17.8 m^2^ (4.8 m wide and 3.7 m long), respectively (Additional file 6: Fig. S3). Plants were grown with 0 g, 8 g, and 15 g N m^−2^ fertilization, along with ammonium sulfate of 4 g N m^−2^ at 8 g N m^−2^ and 15 g N m^−2^ plots and controlled-release fertilizers (LP70-type polyolefin-coated urea, JCAM AGRO Co.) of 4 g N m^−2^ and 7 g N m^−2^ at 8 g N m^−2^ and 15 g N m^−2^ plots, respectively, for basal application. As a top-dressing fertilizer, 2 g N m^−2^ ammonium sulfate was applied twice to both plots during the reproductive stage. LP-70 type fertilizers linearly released 80% of their total N contents for 70 days at 20–30 °C. N fertilizer did not supply to 0 g N m^−2^ plots. Calcium superphosphate (4.37 g phosphate m^−2^) and potassium chloride (6.63 g potassium m^−2^) were applied to all plots on the 3rd to 5th days before transplantation. The weeds, insects and diseases were controlled as required to avoid yield loss. The full-heading date was defined as the time when 40% of the panicles had emerged. Heading dates was 1st August in 2021. Two genotypes of rice plants were considered to have reached yellow, and the plants were harvested in 0 g and 15 g N m^−2^ plots on 20th September and 8 g N m^−2^ plots on 29th September in 2021. During this experimental period, the climate conditions at Kawatabi Field Center are shown in Additional file 5: Table S2.

### Measurement of Relative Light Intensity in Canopy, Dry Weight, and Leaf Area

The characteristics of the rice canopy were investigated at two growth stages: in the early and late ripening stages. The relative light intensity within the canopy at every 20 cm interval from the soil surface to the top of the canopy was measured with photometer (Light Meter Model LI-250, Li-Cor, Lincoln, NE, USA) at the early ripening stage (11th August in 2021). Three sets of samples consisting of four neighboring hills with an average number of panicles for the wild-type and *RBCS*-sense rice plants at 10 DAH in the early ripening (11th August in 2021) and 49 DAH in the late ripening stages (19th September in 2021) were selected from each plot with 15 g N m^−2^ fertilization. The relative light intensity was measured at 5 points between the plants in each layer.

Stratified cutting analyses were performed as described in the report of San-oh et al. (2004) with slight modifications. Rice plants which were one set of four hills were clipped every 20 cm vertically and separated into leaf blades, leaf sheaths and stems, and panicles (Additional file 6: Fig. S4). Three independent experiments were performed. Leaf area was measured with a leaf area meter (AMM-8; Hayashi-denko, Tokyo, Japan). The samples were then dried in a ventilated oven at 80 °C for 3 days to a constant dry weight.

### Measurements of CO_2_ Assimilation Rate of Leaves and Atmosphere CO_2_ Concentrations in the Canopies

The rates of CO_2_ assimilation of flag and penultimate leaves were measured using a portable gas exchange system (LI-6400XT, Li-Cor) at the early ripening stage according to previous reports (Furutani et al. 2020; Suzuki et al. 2021) with slightly modification. The measurement of flag leaves was done on one DHA and that of penultimate leaves was done on four DHA. Measurements were made at a photosynthetic photon flux density of 1500, 1000, 500 and 100 µmol quanta m^−2^ s^−1^, at a leaf temperature of 28–32 °C, a leaf-to-air vapor pressure difference of 1.0–1.2 kPa, relative humidity 70–80% and 400 µmol mol^−1^ of CO_2_ concentration in the leaf chamber of LI-6400XT. After CO_2_ assimilation rate increased and reached the steady state at 1500 µmol quanta m^−2^ s^−1^, photosynthetic photon flux density was changed to obtain 1000, 500 and 100 µmol quanta m^−2^ s^−1^. The measurement time was from 8:00 to 12:00, and outside temperatures were from 26 to 34 °C. Gas exchange parameters were calculated according to the equations proposed by von Caemmerer and Farquhar (1981). The CO_2_ concentration in the vicinity adjacent to the flag leaf (top leaf) of *RBCS*-sense plants in the 0 g and 15 g N m^−2^ plots was continuously monitored with an IRGA (Horiba ASSA-1110, Horiba, Kyoto, Japan) from -26 to 35 DAH as reproductive and ripening periods.

### Physiological and Biochemical Analysis

Dried materials were milled to fine powdered, and then the plant N content was determined using Nessler’s reagent after Kjeldahl digestion. Starch, sucrose and glucose contents in these dried materials were determined by measurements using an F-kit (J.K. International, Tokyo, Japan) according to our previous experiment (Sudo et al. 2014). For Rubisco quantification, we sampled flag and penultimate leaves from the hills different from the hills that were used for stratified cutting in the plots applied with 15 g N m^−2^ fertilization at 3 stages: early ripening (for flag leaves on 5th August in 2021, for penultimate leaves on 9th August in 2021) middle ripening (for flag leaves on 27th August 2021, for penultimate leaves on 31st August in 2021) and late ripening stage (for flag leaves on 19th September in 2021). The Rubisco content was determined spectrophotometrically by formamide extraction of the Coomassie Brilliant Blue R-250-stained subunit bands from the gel (Makino et al. 1994) using calibration curves made with bovine serum albumin (BSA).

### Rice Yield Measurement

The numbers of filled and unfilled spikelets were measured with a counting machine for plant seeds (WAVER IC-VA, VAi, AIDEX Co.). The filled spikelets were separated by submerging the hand-threshed spikelets in a NaCl solution with a specific gravity of 1.06 g cm^−3^, and the ratio of filled spikelets was calculated by comparing the number of filled spikelets with the number of total spikelets. The filled spikelets were then hulled and oven-dried at 80 °C to a constant weight to determine grain dry weight. The weight of the hulled (brown) rice was adjusted to a fresh weight with a moisture content of 0.15 g H_2_O g^−1^. The brown rice yield was calculated by multiplying the grain number per unit land area, the ratio of filled grains and the brown rice weight per grain, which constitutes the ‘yield component’ (Yoon et al. 2020).

### Statistical Analyses

The data are expressed as the mean ± standard error. Student’s *t*-test was performed with Excel (Microsoft). Scatter diagrams and registration lines were created and calculated using Excel (Microsoft). Correlations were tested by Spearman’s rank-order correlation coefficient (*P* value), and covariance analyses were conducted using Excel Tokei (BellCurve).

## Supplementary Information

**Additional file 1: Figure S1** Changes in the dry weights of above-ground sections (shoots), leaf blades, leaf sheaths and stems, panicles, and dead organs of wild-type and RBCS-sense rice plants between 10 DAH in early ripening (A) and 49 DAH in late ripening stages (B) in the plots applied with 15 g N m−2 fertilizer. Mean values ± the standard error of three independent plots are shown. *p < 0.05 between the wild-type and RBCS-sense rice plants using Student’s t-test. The wild-type and RBCS-sense rice plants are represented by black and red bars, respectively. The abbreviations stand as follows: “DAH”; days after heading, “DO”; dead organs, “ER”; early ripening stage, “LB”; leaf blades, “LR”; late ripening stage, “LSS”; leaf sheaths and stems, “P”; panicles, “RBCS-sense”; transgenic rice plants overproducing Rubisco, “S”; shoots (above-ground sections), “Wild”; wild-type rice plant.

**Additional file 2: Table S2** Stem or panicle number of the wild-type and RBCS-sense rice plants at 10 DAH in early and 49 DAH in late ripening stages in plots applied with 15 g N m−2 fertilizer. Mean values ± the standard error of three independent plots, and those of stem numbers at an early ripening and panicle numbers at late ripening stages are shown. *p < 0.05 between the wild-type and RBCS-sense rice plants using Student’s t-test. The abbreviations stand as follows: “ER”; early ripening stage, “LR”; late-ripening stage, “RBCS-sense”; transgenic rice plants overproducing Rubisco, “Wild”; wild-type rice plants.

**Additional file 3: Figure S2** N contents of above-ground sections (shoots), leaf blades, sheaths and stems, and panicles at 10 DAH in early ripening (A) and 49 DAH in the late ripening stages (B) in the plots applied with 15 g N m−2 fertilizer. Mean values ± the standard error of three independent plots are indicated. *p < 0.05 between the wild-type and RBCS-sense rice plants using Student’s t-test. The wild-type and RBCS-sense rice plants are shown by black and red bars, respectively. The abbreviations stand as follows: “ER”; early ripening stage, “LB”; leaf blades, “LSS”; leaf sheaths and stems, “LR”; late ripening stage, “P”; panicles, “RBCS-sense”; transgenic rice plants overproducing Rubisco, “S”; shoots (above-ground sections), “Wild”; wild-type rice plants.

**Additional file 4: Figure S3** Panoramic view of the Isolated Farm for Genetically Modified Plants (Isolated Paddy Field) of the Field Science Center, Tohoku University (Kawatabi Field Center; 38˚44′ N, 140˚45′ E, at 140 m altitude) (A) and planting map in 2021 (B). Experimental paddy fields used in this research are shown in the red frame (A). The abbreviations stand as follows: “RBCS-sense”; transgenic rice plants overproducing Rubisco, “Wild”; wild-type rice plants.

**Additional file 5: Table S2** Climatic conditions in 2021. Climatic conditions at Kawatabi Field Center (38°44′ N, 140°45′ E, at 140-m altitude) from May to October 2021. This covers the rice cultivation period in the experimental paddy field. For “Temp.,” yellow and light blue indicate a change in the average temperature of >1.0°C or <–1.0°C, respectively. For “Sunshine,” yellow and light blue highlights show a period with >120% or <80% of the average sunshine duration, respectively. Gray highlights show a temperature within 1°C of the average temperature and a sunshine duration of 80%–120% of the average. Data on the average weather over the past 30 years at Kawatabi Field Center, Miyagi Prefecture, Japan, are available on the Japan Meteorological Agency (JMA) website (JMA, http://www.data.jma.go.jp/gmd/risk/obsdl/index.php). The abbreviations stand as follows: “A.v.,” average, “Temp.,” temperature.

**Additional file 6: Figure S4** Schematic diagram of stratified cutting method. Rice plants which were one set of four hills were clipped every 20 cm vertically, and separated into leaf blades, leaf sheaths and stems, and panicles. Three independent experiments were performed.

## Data Availability

The datasets supporting the conclusions of this article are included within the article and its additional files.

## Abbreviations

Chl: Chlorophyll
DAH: Days after heading
LAI: Leaf area index
N: Nitrogen
NSC: Nonstructural carbohydrates
*RBCS*: Rubisco small subunit
Rubisco: Ribulose-1,5-bisphosphate carboxylase/oxygenase
