# Supplementary material for: Photosynthetic Enhancement, Lifespan Extension, and Leaf Area Enlargement in Flag Leaves Increased the Yield of Transgenic Rice Plants Overproducing Rubisco Under Sufficient N Fertilization
Source: Rice (N Y). 2022 Feb 9;15:10. doi: 10.1186/s12284-022-00557-5 (PMC8828814; doi:10.1186/s12284-022-00557-5)
Supplement: Supplementary file 1 — Additional file 1: Figure S1 Changes in the dry weights of above-ground sections (shoots), leaf blades, leaf sheaths and stems, panicles, and dead organs of wild-type and RBCS-sense rice plants between 10 DAH in early ripening (A) and 49 DAH in late ripening stages (B) in the plots applied with 15 g N m−2 fertilizer. Mean values ± the standard error of three independent plots are shown. *p < 0.05 between the wild-type and RBCS-sense rice plants using Student’s t-test. The wild-type and RBCS-sense rice plants are represented by black and red bars, respectively. The abbreviations stand as follows: “DAH”; days after heading, “DO”; dead organs, “ER”; early ripening stage, “LB”; leaf blades, “LR”; late ripening stage, “LSS”; leaf sheaths and stems, “P”; panicles, “RBCS-sense”; transgenic rice plants overproducing Rubisco, “S”; shoots (above-ground sections), “Wild”; wild-type rice plant. [file 12284_2022_557_MOESM1_ESM.pdf]

Supplementary File 1

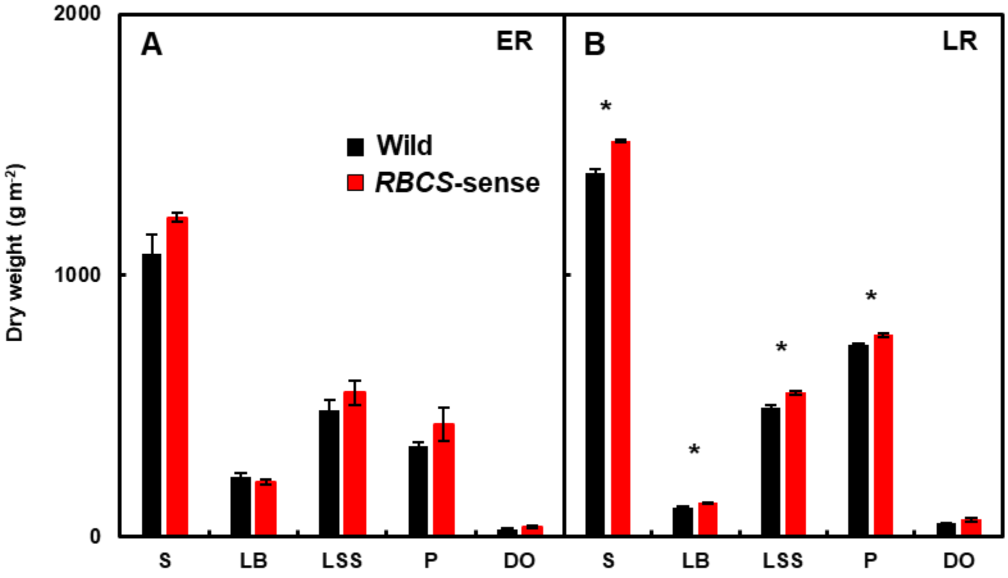

Fig. S1 Changes in the dry weights of above-ground sections (shoots), leaf blades, leaf sheaths and stems, panicles, and dead organs of wild-type and *RBCS*-sense rice plants between 10 DAH in early ripening (A) and 49 DAH in late ripening stages (B) in the plots applied with 15 g N m^{-2} fertilizer.

Mean values ± the standard error of three independent plots are shown. *$p < 0.05$ between the wild-type and *RBCS*-sense rice plants using Student's *t*-test. The wild-type and *RBCS*-sense rice plants are represented by black and red bars, respectively. The abbreviations stand as follows: "DAH"; days after heading, "DO"; dead organs, "ER"; early ripening stage, "LB"; leaf blades, "LR"; late ripening stage, "LSS"; leaf sheaths and stems, "P"; panicles, "*RBCS*-sense"; transgenic rice plants overproducing Rubisco, "S"; shoots (above-ground sections), "Wild"; wild-type rice plant.
