# Supplementary material for: Photosynthetic Enhancement, Lifespan Extension, and Leaf Area Enlargement in Flag Leaves Increased the Yield of Transgenic Rice Plants Overproducing Rubisco Under Sufficient N Fertilization
Source: Rice (N Y). 2022 Feb 9;15:10. doi: 10.1186/s12284-022-00557-5 (PMC8828814; doi:10.1186/s12284-022-00557-5)
Supplement: Supplementary file 2 — Additional file 2: Table S2 Stem or panicle number of the wild-type and RBCS-sense rice plants at 10 DAH in early and 49 DAH in late ripening stages in plots applied with 15 g N m−2 fertilizer. Mean values ± the standard error of three independent plots, and those of stem numbers at an early ripening and panicle numbers at late ripening stages are shown. *p < 0.05 between the wild-type and RBCS-sense rice plants using Student’s t-test. The abbreviations stand as follows: “ER”; early ripening stage, “LR”; late-ripening stage, “RBCS-sense”; transgenic rice plants overproducing Rubisco, “Wild”; wild-type rice plants. [file 12284_2022_557_MOESM2_ESM.pdf]

Supplementary File 2

Table S1

Stem or panicle number of the wild-type and *RBCS*-sense rice plants at 10 DAH in early and 49 DAH in late ripening stages in plots applied with 15 g N m^{-2} fertilizer.

Line	Stem or panicle numbers (m^{-2})	
	ER	LR
Wild	442 ± 35	407 ± 13
RBCS-sense	436 ± 23	398 ± 8

Mean values ± the standard error of three independent plots, and those of stem numbers at an early ripening and panicle numbers at late ripening stages are shown. Statistical analysis was conducted by Student's *t*-test ($p < 0.05$). The abbreviations stand as follows: "ER"; early ripening stage, "LR"; late-ripening stage, "*RBCS*-sense"; transgenic rice plants overproducing Rubisco, "Wild"; wild-type rice plants.
