# Supplementary material for: Photosynthetic Enhancement, Lifespan Extension, and Leaf Area Enlargement in Flag Leaves Increased the Yield of Transgenic Rice Plants Overproducing Rubisco Under Sufficient N Fertilization
Source: Rice (N Y). 2022 Feb 9;15:10. doi: 10.1186/s12284-022-00557-5 (PMC8828814; doi:10.1186/s12284-022-00557-5)
Supplement: Supplementary file 3 — Additional file 3: Figure S2 N contents of above-ground sections (shoots), leaf blades, sheaths and stems, and panicles at 10 DAH in early ripening (A) and 49 DAH in the late ripening stages (B) in the plots applied with 15 g N m−2 fertilizer. Mean values ± the standard error of three independent plots are indicated. *p < 0.05 between the wild-type and RBCS-sense rice plants using Student’s t-test. The wild-type and RBCS-sense rice plants are shown by black and red bars, respectively. The abbreviations stand as follows: “ER”; early ripening stage, “LB”; leaf blades, “LSS”; leaf sheaths and stems, “LR”; late ripening stage, “P”; panicles, “RBCS-sense”; transgenic rice plants overproducing Rubisco, “S”; shoots (above-ground sections), “Wild”; wild-type rice plants. [file 12284_2022_557_MOESM3_ESM.pdf]

Supplementary File 3

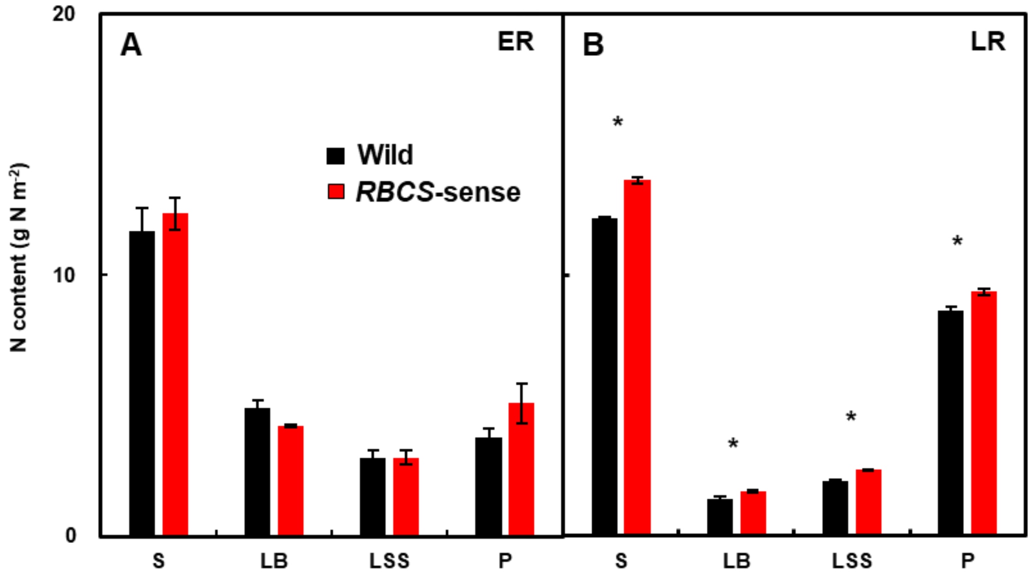

Fig. S2 **N contents of above-ground sections (shoots), leaf blades, sheaths and stems, and panicles at 10 DAH in early ripening (A) and 49 DAH in the late ripening stages (B) in the plots applied with 15 g N m^{-2} fertilizer.** Mean values ± the standard error of three independent plots are indicated. *p < 0.05 between the wild-type and *RBCS*-sense rice plants using Student's *t*-test. The wild-type and *RBCS*-sense rice plants are shown by black and red bars, respectively. The abbreviations stand as follows: "ER"; early ripening stage, "LB"; leaf blades, "LSS"; leaf sheaths and stems, "LR"; late ripening stage, "P"; panicles, "*RBCS*-sense"; transgenic rice plants overproducing Rubisco, "S"; shoots (above-ground sections), "Wild"; wild-type rice plants.
