# Supplementary material for: Photosynthetic Enhancement, Lifespan Extension, and Leaf Area Enlargement in Flag Leaves Increased the Yield of Transgenic Rice Plants Overproducing Rubisco Under Sufficient N Fertilization
Source: Rice (N Y). 2022 Feb 9;15:10. doi: 10.1186/s12284-022-00557-5 (PMC8828814; doi:10.1186/s12284-022-00557-5)
Supplement: Supplementary file 4 — Additional file 4: Figure S3 Panoramic view of the Isolated Farm for Genetically Modified Plants (Isolated Paddy Field) of the Field Science Center, Tohoku University (Kawatabi Field Center; 38˚44′ N, 140˚45′ E, at 140 m altitude) (A) and planting map in 2021 (B). Experimental paddy fields used in this research are shown in the red frame (A). The abbreviations stand as follows: “RBCS-sense”; transgenic rice plants overproducing Rubisco, “Wild”; wild-type rice plants. [file 12284_2022_557_MOESM4_ESM.pdf]

Supplementary File 4

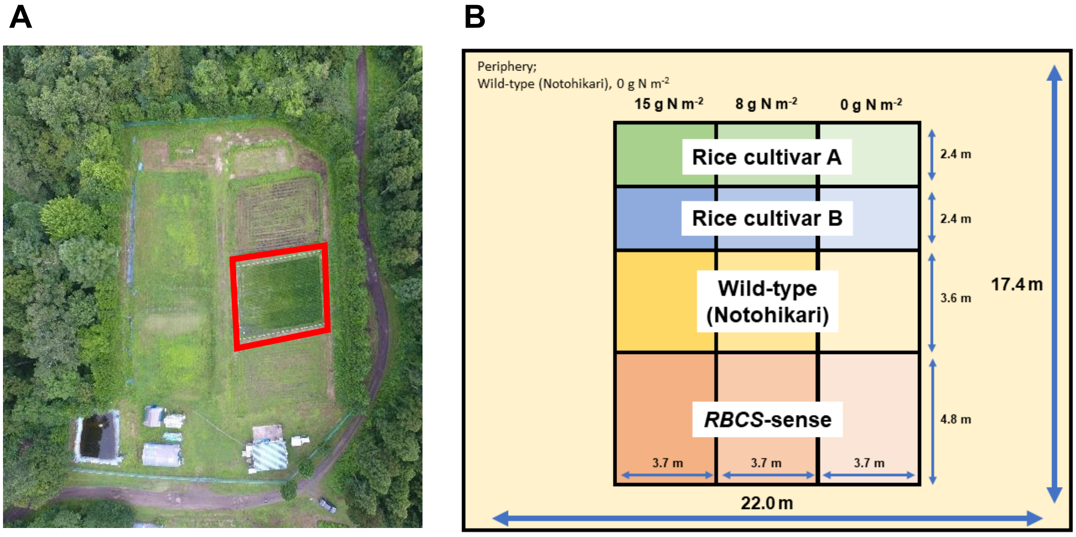

Fig. S3 **Panoramic view of the Isolated Farm for Genetically Modified Plants (Isolated Paddy Field) of the Field Science Center, Tohoku University (Kawatabi Field Center; 38˚44′ N, 140˚45′ E, at 140 m altitude) (A) and planting map in 2021 (B).** Experimental paddy fields used in this research are shown in the red frame (A). The abbreviations stand as follows: "*RBCS*-sense"; transgenic rice plants overproducing Rubisco, "Wild"; wild-type rice plants.
