# Supplementary material for: Photosynthetic Enhancement, Lifespan Extension, and Leaf Area Enlargement in Flag Leaves Increased the Yield of Transgenic Rice Plants Overproducing Rubisco Under Sufficient N Fertilization
Source: Rice (N Y). 2022 Feb 9;15:10. doi: 10.1186/s12284-022-00557-5 (PMC8828814; doi:10.1186/s12284-022-00557-5)
Supplement: Supplementary file 5 — Additional file 5: Table S2 Climatic conditions in 2021. Climatic conditions at Kawatabi Field Center (38°44′ N, 140°45′ E, at 140-m altitude) from May to October 2021. This covers the rice cultivation period in the experimental paddy field. For “Temp.,” yellow and light blue indicate a change in the average temperature of >1.0°C or <–1.0°C, respectively. For “Sunshine,” yellow and light blue highlights show a period with >120% or <80% of the average sunshine duration, respectively. Gray highlights show a temperature within 1°C of the average temperature and a sunshine duration of 80%–120% of the average. Data on the average weather over the past 30 years at Kawatabi Field Center, Miyagi Prefecture, Japan, are available on the Japan Meteorological Agency (JMA) website (JMA, http://www.data.jma.go.jp/gmd/risk/obsdl/index.php). The abbreviations stand as follows: “A.v.,” average, “Temp.,” temperature. [file 12284_2022_557_MOESM5_ESM.pdf]

Supplementary File 5

Table S2

Climatic conditions in 2021

Year		May			June			July			Aug.			Sep.			Oct.	
		Big	Mid.	End	Big.	Mid.	End	Big.	Mid.	End	Big.	Mid.	End	Big.	Mid.	End	Big.	Mid.
A.v.	Temp. (℃)	12.5	13.9	15.7	16.9	17.9	19.3	20.5	21.5	22.6	23.4	22.9	22.1	21.1	19.5	17.2	15.3	13.3
	Sunshine (hr)	61.0	61.1	66.2	58.6	45.1	42.2	38.6	38.1	42.1	46.4	41.5	40.4	37.8	38.0	41.9	42.4	45.4+
2021	Temp. (℃)	13.1	16.1	16.0	19.0	20.2	19.6	20.2	24.1	24.2	26.4	19.9	24.0	18.5	19.6	18.5	17.8	13.8
	Sunshine (hr)	78.5	54.8	66.6	74.2	44.6	42.9	5.3	68.6	41.2	53.9	14.4	35.9	28.6	44.6	54.6	39.0	17.3

Climatic conditions at Kawatabi Field Center (38°44′ N, 140°45′ E, at 140-m altitude) from May to October 2021. This covers the rice cultivation period in the experimental paddy field. For “Temp.,” yellow and light blue indicate a change in the average temperature of >1.0°C or <–1.0°C, respectively. For “Sunshine,” yellow and light blue highlights show a period with >120% or <80% of the average sunshine duration, respectively. Gray highlights show a temperature within 1°C of the average temperature and a sunshine duration of 80%–120% of the average. Data on the average weather over the past 30 years at Kawatabi Field Center, Miyagi Prefecture, Japan, are available on the Japan Meteorological Agency (JMA) website (JMA, http://www.data.jma.go.jp/gmd/risk/obsdl/index.php). The abbreviations stand as follows: “A.v.,” average, “Temp.,” temperature.
