# Supplementary material for: Photosynthetic Enhancement, Lifespan Extension, and Leaf Area Enlargement in Flag Leaves Increased the Yield of Transgenic Rice Plants Overproducing Rubisco Under Sufficient N Fertilization
Source: Rice (N Y). 2022 Feb 9;15:10. doi: 10.1186/s12284-022-00557-5 (PMC8828814; doi:10.1186/s12284-022-00557-5)
Supplement: Supplementary file 6 — Additional file 6: Figure S4 Schematic diagram of stratified cutting method. Rice plants which were one set of four hills were clipped every 20 cm vertically, and separated into leaf blades, leaf sheaths and stems, and panicles. Three independent experiments were performed. [file 12284_2022_557_MOESM6_ESM.pdf]

Supplementary File 6

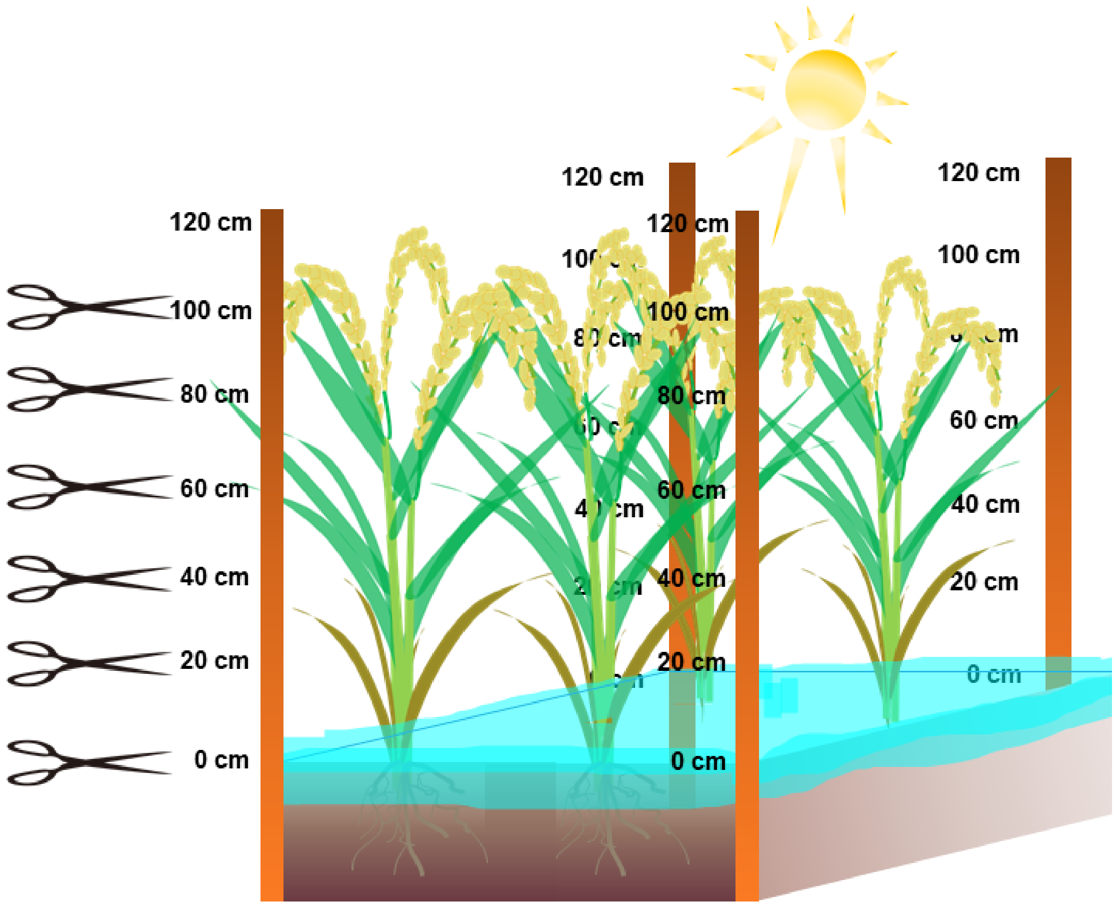

Fig. S4 Schematic diagram of stratified cutting method. Rice plants which were one set of four hills were clipped every 20 cm vertically, and separated into leaf blades, leaf sheaths and stems, and panicles. Three independent experiments were performed.
